# Features of pediatric spontaneous pneumomediastinum: a single-center, retrospective case series

**DOI:** 10.3389/fped.2026.1898720

**Published:** 2026-07-09

**Authors:** Yoshihiko Takano, Mirei Hoshino, Mayuko Fujita, Sakae Iriyama, Kyoko Takayanagi, Nobuhiro Kawakami, Takayuki Okamura

**Affiliations:** Department of Pediatrics, Sakai City Medical Center, Sakai City/Osaka, Japan

**Keywords:** adolescent, body mass index, children, Macklin effect, meteorological factors, spontaneous pneumomediastinum, spontaneous pneumothorax

## Abstract

**Introduction:**

The clinical and epidemiological features of spontaneous pneumomediastinum (SPM) have not been fully characterized, and no previous studies have investigated the association between the onset of SPM and meteorological factors. This study aimed to examine the features of pediatric SPM and to investigate the association between its onset and meteorological factors.

**Methods:**

The medical records of patients aged 1–18 years diagnosed with SPM between January 2012 and December 2023 were retrospectively reviewed. Meteorological data for the same 12-year period were extracted from the Japan Meteorological Agency website. Multivariable logistic regression analysis was performed to evaluate the association between the onset of SPM and monthly meteorological factors.

**Results:**

Overall, 39 SPM cases were identified, including 1 recurrence. Of these, 34 (87.2%) were male with a median age at onset of 15.0 years. The median body mass index of the 26 boys with available data was 19.0 kg/m^2^. The most common presenting complaint at the time of onset was chest pain, which was present in 23 (59.0%) individuals. Statistical analysis revealed a potential association between SPM onset and the monthly average ambient temperature (adjusted odds ratio 1.022; 95% confidence interval 1.010–1.035; *p* < 0.001).

**Conclusions:**

Patients diagnosed with SPM at the authors’ hospital were predominantly adolescent boys with slim builds. We suggest that SPM should be considered as a differential diagnosis in cases of chest pain in slender adolescent boys. No clear association between the onset of SPM and meteorological parameters was identified; further studies using more robust methods are necessary.

## Introduction

Spontaneous pneumomediastinum (SPM) is defined as the presence of free air within the mediastinal interstitium of healthy individuals without any particular trigger such as trauma, surgery, or endotracheal intubation (1). The most likely etiology of SPM is the Macklin effect, in which an increase in intra-alveolar pressure due to body movement (e.g., the Valsalva maneuver) causes alveolar rupture, after which the leaked air reaches the pulmonary hilar region while dissecting along the bronchovascular sheaths, leading to mediastinal emphysema (2). SPM is considered rare in the pediatric population, and its annual incidence rate remains unknown. Previous reports have estimated that SPM occurs in approximately 1 in 8,000 to 1 in 15,000 individuals younger than 18 years of age who present to the emergency department; however, this estimate varies depending on the study population and diagnostic methods used (1). The most common clinical manifestations of SPM at presentation include chest pain, dyspnea, and subcutaneous emphysema. The diagnosis is generally established using imaging modalities such as chest radiography and chest computed tomography (3). Although the prognosis of pediatric SPM is generally favorable as in adults, severe or extensive air leakage may occasionally cause airway compression, resulting in respiratory failure. Furthermore, some cases of SPM may be associated with serious underlying conditions–such as esophageal injury and foreign body ingestion–that require urgent diagnosis and treatment (1). However, these characteristics are based primarily on studies of non-Japanese populations, and clinical and epidemiological features of pediatric SPM among Japanese individuals remain insufficiently characterized; therefore, further investigation is warranted.

SPM is more prevalent among young males and in individuals with slender builds (3–5), and it is more likely to develop following body movement or respiratory diseases such as asthma and pneumonia (1, 3, 6). These features are shared by primary spontaneous pneumothorax (PSP), in which alveolar rupture could be a contributing factor (7–9). Consequently, it has been suggested that SPM and PSP share a common pathophysiological mechanism (10, 11). However, although many studies have examined the association between PSP onset and meteorological factors based on the hypothesis that seasonal changes in atmospheric pressure and temperature contribute to PSP onset (12, 13), no such investigations have been conducted with respect to SPM. As such, the present study aimed to examine the clinical and epidemiological features of pediatric SPM at the authors’ institution, and to investigate the association between the onset of SPM and meteorological factors.

## Materials and methods

### Study design and population

The present study was conducted at Sakai City Medical Center, a municipal hospital located in Sakai City, Osaka Prefecture, Japan. This center serves approximately 6,500 outpatients and 1,600 inpatients ≤ 18 years of age annually. Approximately 81% to 85% of patients attending this hospital reside in Sakai City, which has a population of approximately 140,000 to 150,000 individuals ≤ 18 years of age.

The medical records of patients aged 1–18 years, who attended the authors’ hospital between January 2012 and December 2023, were retrospectively reviewed to identify cases of SPM. This review included only patients for whom the exact date of SPM onset, defined as the onset of patient-reported complaints, could be identified. Patients experiencing pneumomediastinum occurring in the setting of mechanical ventilation, iatrogenic procedures, trauma, or neonatal pneumomediastinum were excluded because these can be considered separate entities. For each identified SPM case, it was confirmed whether the patient had been diagnosed using chest radiography and/or chest computed tomography, and whether free air was present within the mediastinum. Additionally, data regarding patient age, sex, height, weight, body mass index (BMI), date and presumed location of SPM onset, chief complaints at presentation, possible triggers or predisposing factors, diagnostic investigations, associated complications, treatments, and outcomes were also collected.

Because previous studies have reported that SPM is more prevalent among young males and those with slender builds (3–5), the obtained BMI values were plotted separately for boys and girls on the BMI reference curve for Japanese children (14), which covers BMI values from birth to age 17 years and 6 months, to assess whether they were predisposed to a slim body type.

### Meteorological data

Because no previous studies have reported an association between the onset of SPM and meteorological factors, the methods of data collection and analysis employed in this study were exploratory. For the 12-year study period, meteorological data were obtained from the local meteorological institute (Osaka District Meteorological Observatory) on the Japan Meteorological Agency website at https://www.jma.go.jp/jma/index.html. This local institute, which is located approximately 16 km from the authors’ hospital at a comparable altitude, provides data regarding daily average values for various weather parameters, including atmospheric pressure (AP), ambient temperature (AT), percentage of clouds covering the sky (PC), relative humidity (RH), and wind speed (WS). It also provides data regarding daily total values for global solar radiation (GSR), rainfall (RF), and sunshine duration (SD).

Given the rarity of SPM and in light of recent studies examining associations between disease incidence and meteorological factors using monthly meteorological parameters (15–17), monthly data were adopted for the present study. Accordingly, the monthly average values were calculated for 5 parameters (AP, AT, PC, RH, and WS), and the monthly aggregate values for the remaining 3 parameters (GSR, RF, and SD).

To provide an overview of trends in both meteorological parameters and SPM onset, graphs illustrating monthly variations in each parameter throughout the study period were created using the monthly values obtained from the aforementioned calculations. On these graphs, circular markers were plotted to indicate months in which a single SPM event occurred, and larger square markers denoted months with ≥ 2 SPM events.

### Statistical analysis

A case-by-case approach was adopted for enumeration, wherein multiple recurrences within the same patient were considered as independent events. Quantitative variables were expressed as medians and interquartile ranges (IQRs), unless otherwise specified. Differences in meteorological parameters between months with and without reported cases of SPM were investigated. Because the analysis in the current study was unprecedented, the association between SPM onset and each meteorological parameter was initially evaluated using univariable regression analysis, followed by multivariable logistic regression analysis as an exploratory ecological analysis. For patients with recurrent SPM, only the first episode was included in the statistical analyses to maintain independence of observations. Multivariable analysis models were adjusted for the number of SPM cases and combinations of meteorological parameters. The optimal model was selected according to the Akaike information criterion and the Bayesian information criterion. In addition, the goodness of fit of the analytical models selected in the aforementioned manner was evaluated using the Hosmer-Lemeshow test. The presence of multicollinearity was also assessed using variance inflation factor values. Poisson regression analysis, a method for analyzing data on rare events such as SPM, was employed for sensitivity analysis. In order to conduct Poisson regression analysis, an analytical model based on the incidence rates of SPM was required; however, the actual monthly SPM incidence rate in Sakai City was unknown, because data from hospitals in Sakai City other than our institution were not available. As such, Poisson regression analysis was performed using a hypothetical incidence rate. The hypothetical monthly incidence rate was calculated based on the number of SPM cases at the authors’ hospital and monthly population data for individuals aged 1–18 years over the 12-year period, as obtained from the Sakai City website at https://www.city.sakai.lg.jp/shisei/tokei/nenreibetsu/zensikunenrei.html.

Univariable and multivariable Poisson regression analyses were performed using the combination of explanatory variables deemed optimal in the logistic regression analysis, as described above. Statistical analyses were conducted using R statistical software (version 4.3.1) from the R Foundation for Statistical Computing. Differences with *p* < 0.05 were considered statistically significant.

### Ethical approval

Informed consent was obtained via an opt-out approach detailed on the authors’ hospital website. This study was approved by the Institutional Review Board of Sakai City Medical Center (Approval number, 22-323).

## Results

Thirty-nine cases of SPM, diagnosed between January 2012 and December 2023, were identified. The clinical and epidemiological data of these 39 cases are summarized in Table 1, and the complete dataset are available in online Supplementary Table S1.

**Table 1:** Clinical and epidemiological data of SPM cases.

Characteristics	Number	
Sex	n	(%)
Male	34	(87.2%)
Female	5	(12.8%)
Age at SPM onset (years)	median (IQR)	
	15.0	(14.3–17.1)
BMI (kg/m^2^)	median (IQR)	
31 of both sexes*	19.5	(17.5–21.3)
26 males*	19.0	(17.4–20.7)
5 females	20.8	(20.7–21.7)
Season of SPM onset	n	(%)
Spring (Mar–May)	6	(15.4%)
Summer (Jun–Aug)	13	(33.3%)
Autumn (Sep–Nov)	14	(35.9%)
Winter (Dec–Feb)	6	(15.4%)
Presumed place of SPM onset	n	(%)
Indoor	17	(43.6%)
Outdoor	9	(23.1%)
Unknown	13	(33.3%)
Chief complaint	n	(%)
Chest pain	23	(59.0%)
Pharyngalgia	8	(20.5%)
Neck pain	4	(10.3%)
Dyspnea	2	(5.1%)
Others	2	(5.1%)
Trigger factor	n	(%)
Unknown	23	(59.0%)
Physical exercise	9	(23.1%)
Shouting	2	(5.1%)
Others	5	(12.8%)
Diagnostic modalities^†^	n	(%)
Chest x-ray	39	(100%)
Computed tomography	37	(94.9%)
Barium esophagography	1	(2.6%)
Complications^†^	n	(%)
Subcutaneous emphysema	17	(43.6%)
Pneumorrhachis	2	(5.1%)
None	21	(53.8%)

* BMI data is missing in 8 male cases.

† Duplicate cases are included.

BMI, body mass index; IQR, interquartile range; SPM, spontaneous pneumomediastinum.

The median age at onset was 15.0 years (IQR 14.3–17.1). SPM was identified in one 9-year-old child; however, all other cases involved children ≥ 12 years of age. Of the 39 cases, 34 (87.2%) were male (1 recurred at two-year intervals), and 5 were female. The median BMI for 31 cases (patients of both sexes for whom BMI data were available) was 19.5 kg/m^2^ (IQR 17.5–21.3). BMI data were not available for 8 male cases; among the remaining 26 males, the median BMI was 19.0 kg/m^2^ (IQR 17.4–20.7). The 5 females had BMIs of 17.5, 20.7, 20.8, 21.7, and 23.1 kg/m^2^. The BMI values for each case were plotted on a Japanese BMI reference curve according to sex, covering ages up to 17 years and 6 months (14).

**Figure 1:**
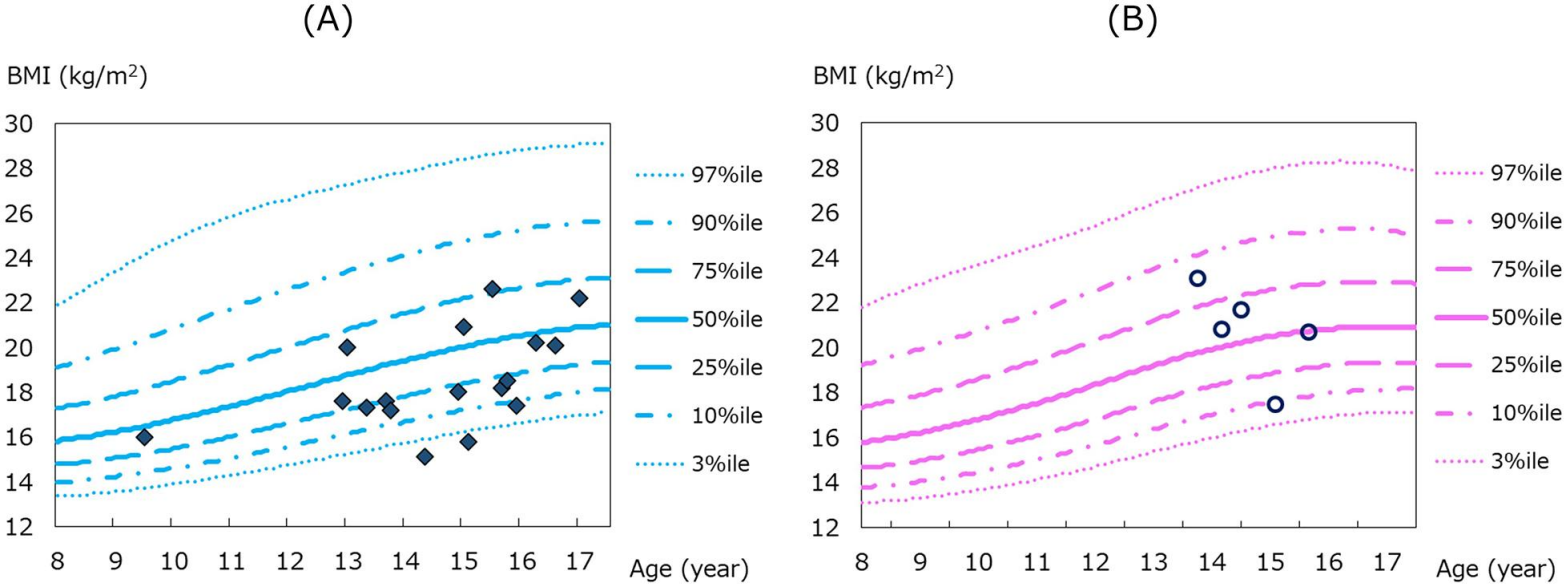
BMI values in each case of SPM were plotted on the BMI reference curve for Japanese children. Only cases of SPM in individuals aged 17 years and 6 months or younger for whom BMI values were available were plotted. **(A)** BMI percentile chart for boys **(B)** BMI percentile chart for girls. BMI, body mass index; %tile, percentile; SPM, spontaneous pneumomediastinum.

As shown in Figure 1, 13 (76.4%) of the 17 male cases whose BMIs could be plotted on the reference curve were below the 50th percentile, and 9 (52.9%) were below the 25th percentile. Among the female cases, only 1 (20.0%) was below the 25th percentile.

Autumn had the highest incidence of SPM, with 14 cases (35.9%), followed by summer, with 13 (33.3%). The presumed locations of onset were indoors in 17 cases (43.6%), outdoors in 9 (23.1%), and unknown in the remaining 13 (33.3%). Of the 39 cases, 23 patients (59.0%) complained of chest pain at the time of onset, 8 (20.5%) complained of pharyngalgia, and others reported symptoms such as neck pain and dyspnea. In 23 cases (59.0%), the trigger factors were unknown, whereas physical exercise and shouting were suspected to be trigger factors in the remaining cases. There were no cases of foreign body aspiration or esophageal rupture due to vomiting. SPM was evaluated using chest radiography in all 39 cases and chest computed tomography in 37 (94.9%). Barium esophagography was performed in only 1 case (2.6%); bronchoscopy was not performed in any case. Subcutaneous emphysema was observed in 17 cases (43.6%), and pneumorrhachis, in which free air is present within the spinal canal, was observed in 2 cases (5.1%). No cases of concomitant pneumothorax or other serious complications were identified. All patients received conservative management and recovered without sequelae.

**Figure 2:**
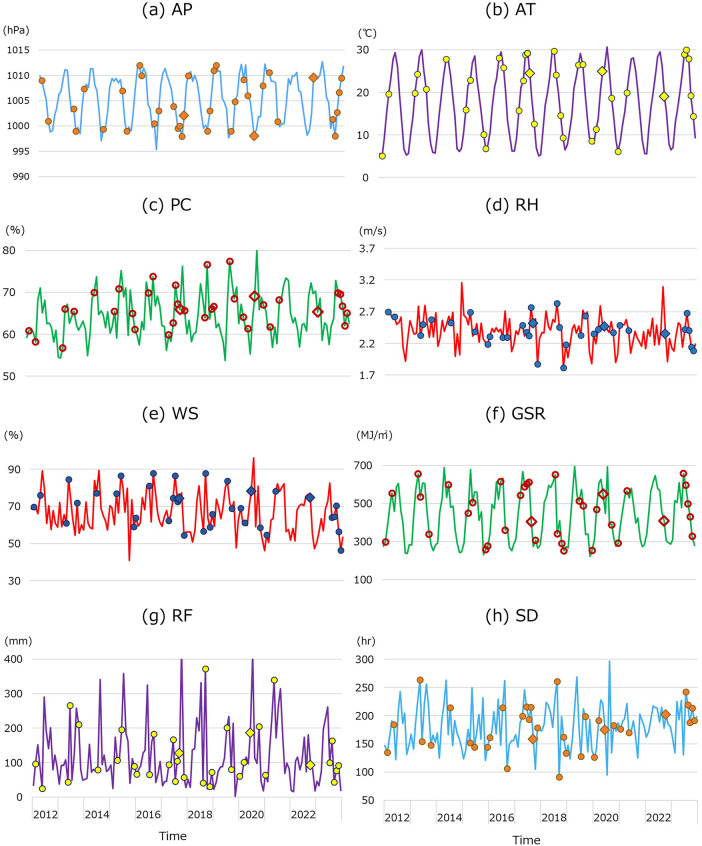
An overview of monthly changes in meteorological parameters from 2012 to 2023 in Osaka, Japan. The lines on the graph represent monthly averages for **(a)** AP, **(b)** AT, **(c)** PC, **(d)** RH, and **(e)** WS, and monthly aggregates of **(f)** GSR, **(g)** RF, and **(h)** SD. Circular markers indicate months with 1 case of SPM. The 3 larger square markers indicate months with 2 SPM cases. AP, atmospheric pressure; AT, ambient temperature; GSR, global solar radiation; PC, percentage of clouds covering the sky; RF, rainfall; RH, relative humidity; SD, sunshine duration; SPM, spontaneous pneumomediastinum; WS, wind speed.

An overview of the monthly variations in meteorological parameters and SPM onset points for each of the 8 parameters over the 12-year period is presented in Figure 2.

Univariable logistic regression analysis revealed that AT and RH had slightly higher odds of developing SPM, with odds ratios of 1.009 (95% confidence interval [CI]: 1.001–1.018; *p* = 0.04) and 1.016 (95% CI: 1.003–1.030; *p* = 0.02), respectively (online Supplementary Table S2). However, no associations were observed with the remaining 6 parameters. In the multivariable analysis, due to the limited number of SPM cases (*n* = 38: 1 recurrent case was excluded from the statistical analysis), an analytical model incorporating 3 explanatory variables was employed. Among all possible combinations of 3 out of the 8 explanatory variables, the combination of AT, RF, and SD yielded the lowest Akaike information criterion and Bayesian information criterion values (online Supplementary Table S3). In the analytical model using these 3 explanatory variables, the Hosmer-Lemeshow test revealed no significant differences, and the variance inflation factor values exhibited no multicollinearity (online Supplementary Table S3); therefore, this model was deemed a good fit. A multivariable logistic regression analysis using this model, which incorporated AT, RF, and SD, revealed that AT had slightly higher odds of developing SPM, with an adjusted odds ratio of 1.021 (95% CI: 1.009–1.033; *p* < 0.001), whereas RF and SD had slightly lower odds, with odds ratios of 0.999 (95% CI: 0.998–0.9999; *p* = 0.04) and 0.997 (95% CI: 0.995–0.999; *p* = 0.01), respectively (Table 2).

**Table 2:** Association between SPM onset and monthly meteorological parameters.

(a) Logistic regression (multivariable) analysis				
	Coefficient	Odds ratio	95% CI	p value
AT (℃)	0.022	1.022	1.010–1.035	< 0.001
RF (mm)	−0.0012	0.999	0.998–0.9998	0.02
SD (hr)	−0.0026	0.997	0.995–0.999	0.02

(b) Poisson regression (multivariable) analysis				
	Coefficient	Risk ratio	95% CI	p value
AT (℃)	0.086	1.090	1.027–1.157	0.004
RF (mm)	−0.0044	0.996	0.990–1.000	0.10
SD (hr)	−0.0098	0.990	0.979–1.001	0.08

AT, ambient temperature; CI, confidence interval; RF, rainfall, SD, sunshine duration; SPM, spontaneous pneumomediastinum.

Among the estimated hypothetical monthly incidence rates of SPM, calculated as described above, the highest monthly incidence was observed in October 2022, with 1.588 cases per 100,000 individuals (online Supplementary Table S4). Univariable Poisson regression analysis based on the hypothetical SPM incidence rates indicated that none of the meteorological factors were associated with an increased risk of developing SPM. (online Supplementary Table S2). In multivariable Poisson regression analysis, only AT demonstrated a significant association with the onset of SPM, with an adjusted risk ratio of 1.086 (95% CI: 1.022–1.153; *p* = 0.01), whereas no association was observed between the onset of SPM and RF or SD (Table 2).

## Discussion

In the present study, we characterized the clinical and epidemiological features of SPM in a cohort of Japanese children. Overall, our findings indicated that SPM was more prevalent among adolescent boys with slender body types. These finding were consistent with those of previous SPM studies conducted in non-Japanese populations (4, 18). No previous studies investigating SPM have evaluated patients’ body types using a BMI reference curve, as was employed in the current study. However, several reports have presented actual BMI values (4, 5, 19). Among these, Abbas PI et al. (19) reported that the median BMI and age of 71 primary SPM patients (without underlying respiratory disease) were 19.7 kg/m^2^ (IQR 17.3–24.3) and 14.5 years (IQR 12.3–16), respectively. In our study, the corresponding values for 29 SPM cases without respiratory disease were 19.5 kg/m^2^ (IQR 17.5–20.9) and 15.0 years (IQR 14.0–17.0), respectively. Thus, our findings closely resembled theirs with regard to the BMI and age of SPM patients without underlying respiratory disease. In this study, there were missing BMI values for 8 male cases, making it impossible to determine whether they were slim or not; however, in terms of clinical features other than body type—namely, chief complaints, complications, and treatments—there were no notable differences compared to other SPM cases (Supplementary Table S1). When focusing on the symptoms at onset, among patients with SPM in the study by Noorbakhsh KA et al. (20) the most frequently presenting symptom was chest pain, observed in 120 of 183 patients (66%), which is comparable to the results of our study (59.0%). Previous reports of SPM have similarly identified chest pain as the most common presenting symptom at onset (5, 19, 21). Therefore, we suggest that SPM should be considered as a differential diagnosis in cases of chest pain in slim adolescent boys, despite its rarity in the pediatric population.

In the present study, both analytical models consistently demonstrated that a higher monthly average AT was associated with an increased likelihood of SPM occurrence. For individuals with recurrent SPM, only the first episode was included in the statistical analysis. Similar results were obtained when recurrent episodes were treated as separate cases (data not shown). Although a statistically significant difference was observed, these results should be interpreted with caution. Given the limited number of SPM cases, the analytical model using multiple meteorological parameters is very fragile and may be overfitting or providing unstable estimates. Furthermore, despite a statistically significant association between SPM onset and monthly average AT, the effect size is very small and does not necessarily indicate a clinical relevance. Moreover, changes in AT may influence physical activity levels and patterns of exposure to environmental factors; therefore, it is important to recognize that residual confounding may have affected these results. Accordingly, the findings of this study should be considered hypothesis-generating rather than evidence of a definitive association. Nevertheless, these results are suggestive because they are consistent with the observed seasonal pattern in the study by Kim KS et al. (4), in which 28 (43.8%) out of 64 cases of SPM occurred during the summer, when temperatures rise in South Korea. Although the association between the onset of SPM and meteorological factors has not been previously investigated, numerous studies have examined the association between the onset of PSP and meteorological parameters. Despite numerous reports, no conclusive consensus regarding this association has been reached to date (13). However, a recent meta-analysis by Marx T. et al. (22) posited that higher average temperatures are associated with an increased incidence of PSP. This trend is similar to that observed in our analysis of SPM cases, suggesting that SPM and PSP may share a common etiology. One potential mechanism by which higher AT increases the risk of SPM or PSP development is the activation of transient receptor potential vanilloid 1 receptors on pulmonary C-fibers in response to elevated temperatures, resulting in bronchoconstriction and coughing, which may subsequently lead to alveolar rupture (23, 24). Moreover, indirect effects may also be involved. These include increased physical activity associated with higher AT, which could contribute to exercise-induced alveolar rupture, as well as altered exposure to environmental factors, such as elevated atmospheric ozone concentrations, that accompany rising temperatures. Exposure to ozone can trigger asthma exacerbations and increase susceptibility to respiratory infections, thereby increasing the risk of developing SPM (25, 26). In any case, it is presumed that both SPM and PSP are triggered by air leaks resulting from the rupture of vulnerable alveolar structures, such as bullae or blebs. A recent study reported that while most pulmonary bullae are located immediately beneath the visceral pulmonary pleura, a subset occurs intrapulmonarily (27). We speculate that SPM develops when these intrapulmonary bullae rupture, subsequently triggering the Macklin effect, and that the difference in incidence between SPM and PSP may be partially attributable to differences in the anatomical distribution of bullae within the lungs. To elucidate the relationship between the onset of SPM and meteorological factors and to clarify its underlying etiology, the accumulation of additional SPM cases and relevant data is needed.

The significance of the present study is that this is the first case series to describe the clinical and epidemiological features of pediatric SPM in a cohort of the Japanese population and to investigate the association between the onset of SPM and meteorological factors. Considering that recent research has increasingly focused on the association between diseases and climate change (28–30), our study may provide novel insights into the role of meteorological factors in the onset of SPM.

The current study has several limitations. First, it employed a single-center, retrospective case series design with a limited sample size. Second, as the upper age limit of the BMI reference curve for Japanese children is 17 years and 6 months, we were unable to evaluate whether the 17-year-old (*n* = 4) and 18-year-old (*n* = 5) patients with SPM would be considered to have a slender body type. Third, indoor-onset SPM may be influenced by air conditioning use. Although a substantial proportion of cases in this study occurred indoors (17 of 39 cases), no information regarding air conditioning use was available for any of these cases. Fourth, the statistical analyses performed in this study were highly exploratory. Given the small sample size and inadequate adjustment for potential confounding factors, the statistical models were susceptible to instability, and the findings should therefore be interpreted with considerable caution. Finally, because this study was conducted within a Japanese population, due to regional differences in healthcare-seeking behavior, living environments, and ethnic characteristics, the findings of this study cannot be extrapolated to other populations.

## Conclusions

This 12-year survey conducted at the authors’ hospital revealed that SPM was more prevalent among slender adolescent boys, a trend also observed within Japanese children. In line with previous reports, the most frequently reported presenting complaint at the onset of SPM was chest pain. Therefore, although rare, we suggest that SPM should be considered in the differential diagnosis of chest pain in slender adolescent boys. With regard to the association between SPM onset and meteorological factors, multivariable logistic regression analysis revealed a potential association between SPM onset and higher monthly average AT, with similar results confirmed by sensitivity analysis. However, these results do not demonstrate a definitive relationship; instead, they serve as a basis for generating future hypotheses because of the limited number of cases, the exploratory nature of the analytical model, and potential confounding factors. Furthermore, because ecological and social environments differ across populations, the findings of this study may not be generalizable to other racial groups or geographic regions. To elucidate the role of meteorological factors in the onset of SPM, additional studies employing robust epidemiological methods across multiple centers are warranted.

## Data Availability

The original contributions presented in the study are included in the article/Supplementary Material, further inquiries can be directed to the corresponding author.
